# Home blood pressure data visualization for the management of hypertension: designing for patient and physician information needs

**DOI:** 10.1186/s12911-020-01194-y

**Published:** 2020-08-18

**Authors:** Richelle J. Koopman, Shannon M. Canfield, Jeffery L. Belden, Pete Wegier, Victoria A. Shaffer, K. D. Valentine, Akshay Jain, Linsey M. Steege, Sonal J. Patil, Mihail Popescu, Michael L. LeFevre

**Affiliations:** 1 Department of Family & Community Medicine, University of Missouri-Columbia, Columbia, MO USA; 2 Department of Family & Community Medicine, University of Toronto, Toronto, Ontario Canada; 3 Temmy Latner Centre for Palliative Care, Sinai Health System, Toronto, Ontario Canada; 4 Lunenfeld-Tanenbaum Research Institute, Sinai Health System, Toronto, Ontario Canada; 5 Department of Psychological Sciences, University of Missouri-Columbia, Columbia, MO USA; 6 Health Decision Sciences Center, Massachusetts General Hospital, Boston, MA USA; 7 Harvard Medical School, Boston, MA USA; 8 Department of Electrical & Computer Engineering, University of Missouri-Columbia, Columbia, MO USA; 9 School of Nursing, University of Wisconsin-Madison, Madison, WI USA; 10 Department of Health Management & Informatics, University of Missouri-Columbia, Columbia, MO USA

**Keywords:** Data visualization, Electronic health record, Home blood pressure monitoring, Hypertension

## Abstract

**Background:**

Nearly half of US adults with diagnosed hypertension have uncontrolled blood pressure. Clinical inertia may contribute, including patient-physician uncertainty about how variability in blood pressures impacts overall control. Better information display may support clinician-patient hypertension decision making through reduced cognitive load and improved situational awareness.

**Methods:**

A multidisciplinary team employed iterative user-centered design to create a blood pressure visualization EHR prototype that included patient-generated blood pressure data. An attitude and behavior survey and 10 focus groups with patients (*N* = 16) and physicians (*N* = 24) guided iterative design and confirmation phases. Thematic analysis of qualitative data yielded insights into patient and physician needs for hypertension management.

**Results:**

Most patients indicated measuring home blood pressure, only half share data with physicians. When receiving home blood pressure data, 88% of physicians indicated entering gestalt averages as text into clinical notes. Qualitative findings suggest that including a data visualization that included home blood pressures brought this valued data into physician workflow and decision-making processes. Data visualization helps both patients and physicians to have a fuller understanding of the blood pressure ‘story’ and ultimately promotes the activated engaged patient and prepared proactive physician central to the Chronic Care Model. Both patients and physicians expressed concerns about workflow for entering and using home blood pressure data for clinical care.

**Conclusions:**

Our user-centered design process with physicians and patients produced a well-received blood pressure visualization prototype that includes home blood pressures and addresses patient-physician information needs. Next steps include evaluating a recent EHR visualization implementation, designing annotation functions aligned with users’ needs, and addressing additional stakeholders’ needs (nurses, care managers, caregivers). This significant innovation has potential to improve quality of care for hypertension through better patient-physician understanding of control and goals. It also has the potential to enable remote monitoring of patient blood pressure, a newly reimbursed activity, and is a strong addition to telehealth efforts.

## Background

Hypertension affects 70 million US adults and is a major risk factor for morbidity and mortality, contributing to heart disease, stroke, and chronic kidney disease [1]. Nevertheless, only 48% of US adults with hypertension have their blood pressure controlled, and hypertension control is even worse if using new American Heart Association guidelines [2, 3]. Given broad awareness of the disease and its complications, readily available clinical and home monitoring, and multiple treatment options, why is blood pressure still uncontrolled in half of those diagnosed with hypertension?

Clinical inertia may account for gaps in care in the treatment of hypertension. Clinical inertia is a lack of treatment initiation or intensification despite objective evidence that disease is not controlled [4–6]. At any particular office visit, there is only a 13% probability of treatment intensification for uncontrolled hypertension [6]. An in-depth examination of clinical inertia in the pursuit of blood pressure control showed that clinical uncertainty about the representativeness of any one clinic blood pressure in characterizing overall blood pressure control was a prominent reason that physicians do not act to intensify therapy [7]. Blood pressure naturally rises and falls based on several factors including stress and activity level, so variation is common [8, 9]. These natural fluctuations in blood pressure cause uncertainty for patients as well, as shown in our own research [10], and also persuasively described in the New York Times essay “Blood Pressure, a Reading With a Habit of Straying” [11]:

“I decided to check my blood pressure with a home monitor before a coming physical examination. The first night, I was startled to find that my systolic pressure was a scary 137. The next night, it was only 117. The next morning, before I saw my doctor, it was a terrifying 152. At the doctor’s office, it was 150. I measured it again that night, and it had plummeted to 110.”

Because blood pressure variability adds to uncertainty for both the patient and physician, data visualization tools that can promote better patient and physician understanding of the data can improve the quality of patient-physician negotiations about control and treatment as part of hypertension management [7, 11]. For this reason, our team elected to create a design that could be shared by patients and physicians during a time-limited visit.

**Fig. 1:**
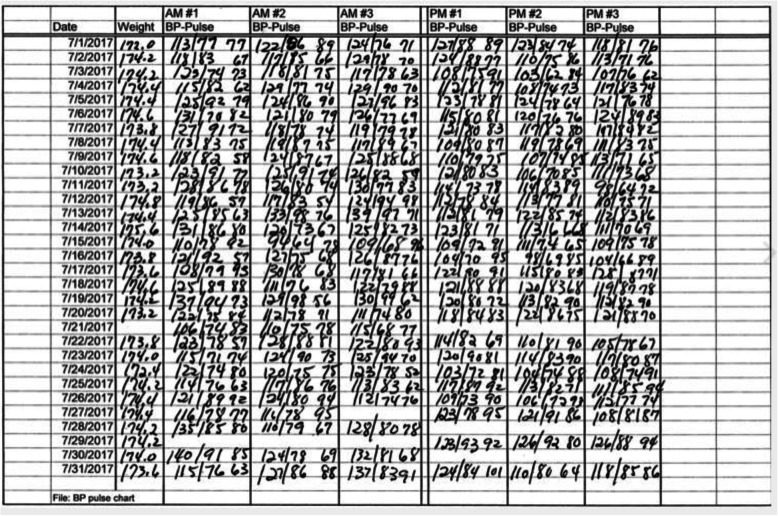
Patient supplied home blood pressure data—detailed and abundant

Home blood pressures may contribute to the variable data problem but are nevertheless important. Several large studies reveal that home blood pressure measurements predict cardiovascular outcomes as well or better than clinic blood pressure measurements, even after accounting for clinic blood pressure [12–18]. Although we aspire to use these patient-generated data and engage patients in their care, home measurements can increase physician cognitive load, as patients frequently bring home blood pressure data to clinicians on pieces of paper during time-limited outpatient visits [19]. This form of data presentation does not fit well with the physician’s electronic workflow, which in our experience typically leads to less than ideal handling of this information, failing to honor the effort of the patient in collecting these data (Fig. 1). While integration of home blood pressure into clinical care is an important goal, achieving integration can be difficult and elusive [19]. Better information display may support physician-patient shared decision making through improved situation awareness and more productive interactions between care teams and patients [20–22]. Finding a way to meaningfully represent both home and clinic blood pressure data for physicians and patients, in a manner that meets their information needs, will be a significant advance that allows us to finally integrate home blood pressure into clinical care, translating evidence to practice.

Well-designed graphs based on principles of human cognition and visual perception can “emphasize relationships, focus interest, save time in analyzing data, help recall, uncover previously hidden facts and break down the language barrier,” [23] as well as improve accuracy for clinical data [23–28]. Data visualizations, particularly icon array and pictographs, may facilitate communication about risk [29, 30]. In some cases, effective graphs may improve clinical outcomes; a small Japanese clinical trial demonstrated that a home blood pressure monitor graphic display was associated with better blood pressure control [31]. However, while literature and principles of cognition can guide design of effective graphs, ineffective graphs abound [28, 32]. A review of Fortune 500 company annual reports revealed that half of them contained inappropriately constructed graphs [33]. Our team’s experience viewing multiple EHRs reveals that EHR graphs are often similarly ineffective [34]. In an effort to improve on the current state, we identified the information needs of patients and physicians for blood pressure data in hypertension (self-)care. We describe our user-centered design process to address these needs, including initial and confirmatory evaluation of the data visualization.

## Methods

### Approach

To identify and satisfy patient and physician information needs for hypertension management, with special attention to the growing evidence for including home blood pressure data, we employed a user-centered design process to incorporate patient home blood pressure data into the clinical EHR and improve data visualization for the care of hypertension. User-centered design is an iterative process that involves understanding user information needs for their work, designing for those needs using known design principals, analyzing if needs are efficiently met by the design, and iterating design with further understanding of needs [34–37].

### Interface design

To create initial candidate designs, our multidisciplinary team used a deep dive approach, incorporating principles of data visualization, graphic and health literacy, human factors, health psychology, the tenets of the Chronic Care Model, and usability best practices, as well as the views of two important user groups, patients and their primary care physicians [38–40]. Our team brought together experts from diverse disciplines and multiple institutions, including Family Medicine, Human Factors Engineering, Informatics, Psychology, Computer Science and Public Health. Foundational underpinnings of these diverse disciplines guided our team’s work to dually design for patients and physicians, creating a blood pressure data visualization that might promote a shared understanding of blood pressure data and trends, and facilitate decision making between patients and physicians, especially during the primary care patient visit [41, 42].

**Fig. 2:**
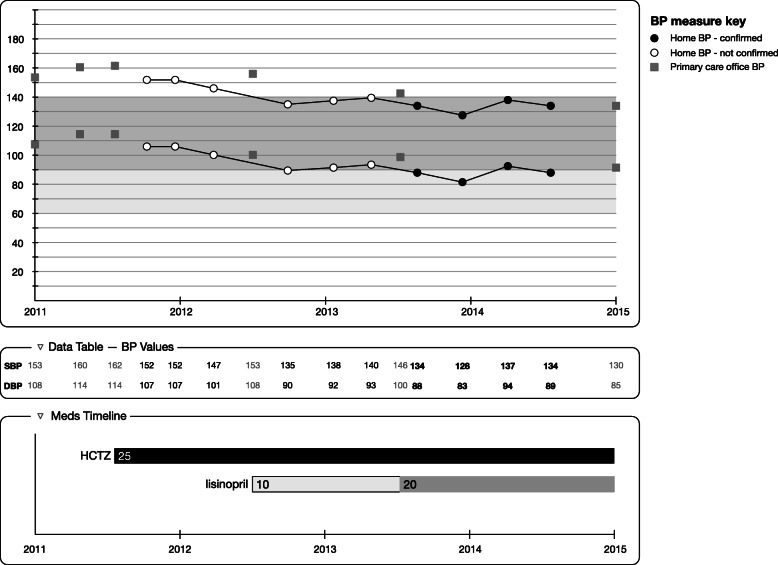
Early design—grayscale, with data table and medication timeline, lines connecting home BPs

Informed by known visual display concepts and evidence from our literature review, we first designed several candidate data visualizations that included a data table of BP values, and an aligned medication timeline (Fig. 2) [23–37, 42]. Concurrently and in parallel, a series of online cognitive perceptual experiments with patients with hypertension also informed our design, including a smoothing algorithm to improve patient judgments about variable blood pressure data [10]. The conduct and results of online perceptual experiments are detailed elsewhere [10]. We concluded iterations when our designs met the user identified information needs, with satisfaction of needs dually determined by focus group participant feedback and multidisciplinary team judgment that the design met expressed needs.

### Patient and physician focus groups

**Fig. 3:**
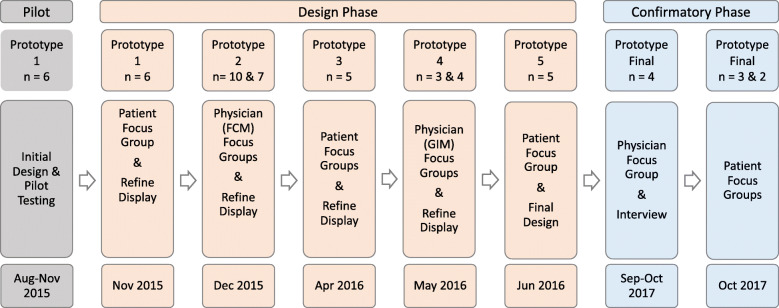
Project design phases: Pilot Phase (grey), Design Phase (peach), Confirmatory Phase (blue). Rounds 2 and 4 of the Design focus groups and Confirmatory Patient round each had 2 focus groups, for a total of 10 Design and Confirmatory focus groups (excluding the pilot focus group)

**Fig. 4:**
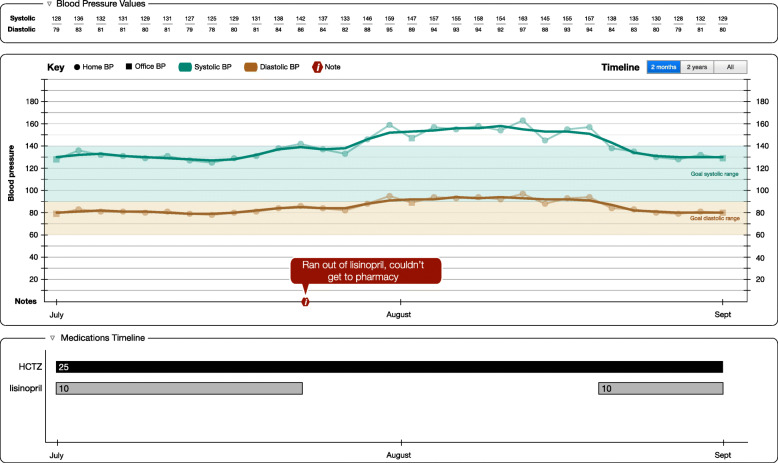
Final prototype— color, with data table and medication timeline, user annotations, lines connecting both home and office BPs

We iteratively tested and refined the visualization in a series of 7 formative design focus groups, with 5 iterations to reach the final prototype (Fig. 3). Design phase focus groups alternated between patient and physician participants, with separate focus groups for patients and physicians (Fig. 3). After iterating to a final prototype (Fig. 4), we conducted three confirmatory focus groups and a key informant interview as a member check. Confirmatory focus groups included participants from the initial patient and physician formative focus groups, with review of the final prototype data visualization for fit with their needs as a member check as well as questions about how they would use the new visualization in their hypertension decision making. We sought to include 5–7 participants in each focus group [43].

#### Participants and recruitment

Family medicine and general internal medicine physicians and their patients age 18 years and older with a diagnosis of hypertension were recruited from eight community-based practices. Prior to the focus groups, we educated physicians about the evidence for home blood pressure use in clinical care in a voluntary grand rounds-type seminar. Physician participants were emailed with information about the opportunity to participate; we conducted focus groups at times and places convenient for the physicians, such as lunchtime in the practice conference room.

Patient participants were patients of these practices age 18 and above with a diagnosis of hypertension. We used the EHR hypertension registry to create lists of patients, asked their physicians to eliminate those with cognitive impairment or serious mental health diagnoses, and mailed recruitment letters to remaining patients asking them to call the coordinator to enroll. The study coordinator confirmed hypertension diagnosis, screened for cognitive impairment, and described the focus group procedure. Eligible patients were invited to the focus group. At the start of the focus group, patients were presented with a waiver of documentation of consent, including both the written waiver and a verbal discussion of the study focus group procedures. Patients were given the opportunity to ask clarifying questions about the study to confirm understanding and withdraw if they preferred. Patients received $30 fair compensation for time and travel. A similar consent process occurred at the beginning of physician focus groups. The University of Missouri Health Sciences Institutional Review Board reviewed and approved this study and its human subjects’ participation and consent process.

#### Focus groups conduct

All physician and patient participants completed a pre-focus group survey to characterize sample demographics and typical data collection, data use, and decision making based on clinic and home blood pressure readings. Beyond characterizing sample demographics, the short attitudes and behaviors survey addressed research questions (see Table 1) that benefited from a quantitative characterization. For example, patients were asked to report on frequency of home blood pressure measurement behaviors and physicians were asked to report how they most often interpreted and recorded home blood pressure data supplied on paper.

**Table 1:** Focus group research questions

Design Phase			Confirmatory Phase
Representation of BP Data	Clinical Guidelines and Goal Ranges	Supplemental Features	Final Prototype Evaluations
How should home and clinic blood pressures be represented in graphic form?	How can we represent different blood pressure goals consistent with clinical guidelines?	What type of linguistic summarization would be useful to physicians and patients?	After we design these features, are they useful to patients and physicians?
How should home blood pressures be distinguished from clinic blood pressures?	Can we make goals customized to the individual patient?	How can the medication history be presented?	Does the visualization meet patient and physician information needs?
How can we effectively summarize numeric and graphic data?	How can we represent the rationale for individualized goals?	How should we include data about whether and when the home blood pressure monitor was validated against clinic blood pressure?	Will they encourage patient education, activation?
		Should a hide/reveal function be used for home blood pressures?	

Experienced focus group facilitators SMC and RK led the focus groups; at least two other authors observed and recorded field notes for each focus group. Focus group data collection also included written comments by participants on a series of paper versions of the visualizations. Focus group questions and prompts were informed by research questions (Table 1) and were designed to elicit broad feedback regarding usability and preferences.

Participants were presented with candidate designs, with iteration of designs between each focus group. Data visualizations presented in focus groups varied in both 1) design element options and 2) the pattern and trend of blood pressures to represent common blood pressure “stories”, e.g. increasing blood pressure trend, addition of new medication and resulting decrease in blood pressure, or a gap in medication adherence associated with blood pressure rising and then returning to goal. Because we were aiming for an intuitive design, focus group participants were challenged to construct meaning from the data visualizations with little to no orientation from the facilitators and to construct stories about the patient represented in the data visualization. For example, the visualization in Fig. 4 was used during a confirmatory focus groups with patients. Participants were asked to “First take a moment to view this image and jot down a few notes. We’d like you to describe how this patient is doing managing their hypertension.” After discussing impressions participants then were asked: “A doctor and patient view this image during a clinical visit. Imagine and then describe that conversation to the group”. Physician participants were also asked to use the visualization in role-plays discussing blood pressure results with a patient in an effort to help us understand any barriers/advantages to using these visualizations during patient visits.

### Analysis

We analyzed patient and physician surveys using descriptive statistics to examine demographics, attitudes and behaviors about blood pressure (self-) management. Analysis of focus group qualitative data took place in three phases: an immediate team debrief, preliminary rapid qualitative analysis immediately following each focus group, and final thematic analysis of compiled data. Rapid qualitative analysis is a process where researchers review transcripts and identify key aspects of the data answering research questions relevant to the iterative design process. We used this method after each focus group to quickly identify participant responses to design features, to confirm our designs or identify a need to iterate design features, and to quickly develop a preliminary understanding of remaining unaddressed patient/physician information needs and preferences [44–46]. Our final qualitative analysis phase was a more comprehensive and traditional thematic analysis [47]. Our approach to thematic analysis included a deductive, realist approach to the data using a theoretical frame to identify key themes across the dataset in response to predefined research questions. Both RJK and SMC are experienced with qualitative research, with RJK a family physician researcher and SMC a long-time project director with a public health, diversity, and patient needs perspective [48–53]. First, researchers RJK and SMC became familiar with the data by reviewing and checking accuracy of verbatim transcripts. Next, using Dedoose©, RJK and SMC individually coded transcripts and then worked together to develop a coding framework based on research questions (Table 1), then conducted consensus coding, and held frequent meetings throughout the process to identify emerging themes and ensure accurate representation of the data [54]. Findings were organized into main and subthemes and shared with the larger team for reaction and refinement. Final names and descriptions were established representing the story of the themes. The analysis of both survey and focus group data (both spoken and written comments) as well as the examination of two stakeholder perspectives, patients and physicians, facilitated convergence and triangulation of these data for the investigators. Finally, our confirmatory focus groups served as member checks.

## Results

### Survey

Focus group participant demographics are reported in Table 2. Most patients (75%) took blood pressure at home, but only half shared that data with physicians. Only two patient participants were aware that their doctor graphed the blood pressure measures using the existing EHR graphing feature. Among physicians, 92% felt graphing blood pressure data was helpful although less reported using graphs in their work. All physicians indicated receiving measures in paper form; 75% via patient portal, and 50% by phone. Most typically, patient home blood pressure data was recorded as a gestalt estimate of the average recorded in a clinical note (*88%*).

**Table 2:** Focus group patient and physician participant characteristics

Characteristics^a^	Patients	Physicians
*N*	16	24
Gender—% (*N*)		
Female	62 (10)	33 (8)
Male	38 (6)	67 (16)
Age—*M (SD)*	59 (17.6)	48 (13.6)
Race—% (*N*)		
White	88 (14)	92 (22)
Black / African American	6 (1)	4 (1)
Other	6 (1)	4 (1)
Ethnicity—% (*N*)		
Latino / Latina	0	0
Education (patients—% (*N*)		
Some college or greater	62 (10)	
High school or GED	19 (3)	
Less than high school	19 (3)	
Years in practice (physicians—% (*N*)		
Less than 5 years		29 (7)
6–20 years		33 (8)
21–30 years		21 (5)
More than 30 years		17 (4)
Attitudes—% (*N*)		
Patient beliefs about use of their home blood pressure		
Physician has nurse/clinic staff person to enter into my record	13 (2)	
We talk about it during my visit	38 (6)	
I do not believe they do anything with the information	7 (1)	
I don’t know / Other	13 (2)	
Not applicable / No response	33 (4)	
Physician beliefs about graphing blood pressure data		
Not at all or not very helpful		4 (1)
Somewhat helpful		29 (7)
Very or extremely helpful		63 (15)
No response		4 (1)
Behaviors—% (*N*)		
Patient takes home blood pressure	75 (12)	
Patient records home blood pressure		
No	25 (3)	
On paper	50 (6)	
Special cuff	25 (3)	
Physician recording of home blood pressure into EHR (all that apply)		
Upload or dictate all values into my clinical notes		25 (6)
Summarize the range or average in my clinical note		58 (14)
Scan the data into patient chart		25 (6)
Get help from other profession staff or nurse		25 (6)
Do not input the data into the electronic record		8 (2)
Other		8 (2)
Physician shares view of patient blood pressure data in EHR		
Sometimes / Often / Always	50 (8)	50 (12)
Rarely / Never	31 (5)	46 (11)
I don’t know / No response	19 (3)	4 (1)
Physician graphs patient blood pressure during visit		
Sometimes / Often / Always	13 (2)	50 (12)
Rarely / Never	31 (5)	46 (11)
I don’t know / No response	19 (3)	4 (1)
Physician uses of home blood pressure data (all that apply)		
Review overall control		92 (22)
Determine the need for treatment		88 (21)
Encourage dialog and communication		83 (20)
Encourage shared decision making		88 (21)
Finalize a treatment plan		83 (20)

^a^Response options included additional categories. Only those reported by participants are included here

### Qualitative results

Our rapid qualitative analysis method, occurring after each focus group, informed our iterative design process by identifying and clarifying information needs for physicians and patients, stimulated design iterations to satisfy those needs, and allowed us to check if participants indicated that our design iterations satisfied those needs. Identified needs and design elements to satisfy those needs are presented in Table 3. Our subsequent, more in-depth thematic analysis revealed the following themes:

**Table 3:** Identified information needs and design elements to address those needs

Identified Information Need^a^	Design Element
Distinguish systolic from diastolic data points	Color differentiation
Visualize clinic home blood pressure data	Include both on visualization
Differentiate clinic and home data	Different symbols for sources
Contextual information about lifestyle and clinical events	Annotations
Relationship between medication and blood pressure	Blood pressure graph stacked with medication timeline
Understanding of blood pressure goals	Shaded goal ranges, default to those appropriate for patients age and comorbidity
Visualize out of range values	Shaded goal ranges were deemed sufficient, several ways of further highlighting out of range values were rejected by both groups
Customizable goal ranges	Customization using radio buttons corresponding to common guidelines
See the raw data	Include stacked data table
Patient burden for data entry	Automated data upload from device
Understand variable blood pressure data	Lowess smoothing line
Understand flow of data to care team	Future design of workflow adaptations for comprehensive hypertension care

^a^ All identified needs were expressed by both physicians and patients with the exception of customizable goal ranges, which was identified solely by physicians

#### Data visualization to enable engaged, activated patients and prepared, proactive physicians

Focus group findings highlight the importance of accurate, practical, and time-relevant information that promotes understanding of the blood pressure story so that patients are engaged and activated and physicians are equipped with the information they need to be prepared and proactive, as suggested by the Chronic Care Model [38, 39]. Both patient and physician participants indicated easily understanding the visualization (Figs. 3 and 4). In cases of confusion, a simple explanation from the facilitator was sufficient. Patients and physicians agreed that both home and clinic blood pressure measures should be included on the data visualization and intuited home blood pressures should be emphasized for general accuracy and to encourage patient engagement. Patient responses indicated visualizations created engagement with blood pressure data and a feeling of preparation and readiness to act, an idea echoed by physicians.

“It [data visualization] would make me feel like if it’s important enough to her to bring it up to me, it ought to be doggone important to me and I should pay close attention to it … I mean, blood pressure is the silent killer...I need to know how I’m doing, where I stand, ‘Girl, you are in trouble, you’re on a collision course. You need to make some changes. Snap out of this, or we’re gonna be increasing meds or adding some.’”

— Patient, Design Round 5

“I think it’s useful to know the values of home versus office and how we’re making decisions on that … I think it will incentivize the patient to do [home monitoring] because we’re making treatment decisions on that … So I think that’s therapeutic.”

— Physician, Design Round 4

**Fig. 5:**
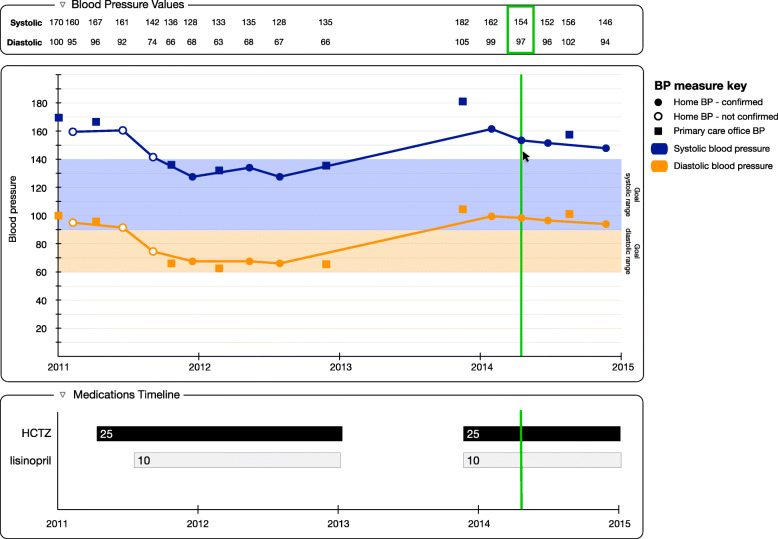
Annotations in graph area, with details in sidebar

Patients and physicians advocated for the addition of contextual information to aid in decision-making. Examples of such information include behavioral/lifestyle change, weight, stressors, and significant clinical events. For this reason, we designed also for the ability to annotate the visualization with this contextual information (Fig. 5).

“Maybe I would reflect a little bit about my lifestyle, you know … but I would reflect back on oh, yeah, that’s right, I was good and watching my salt intake and it’s, you know, kind of relaxed a little bit or, or maybe forget to take your medications, you know.”

— Patient, Confirmatory Round

“Well, the whole system sort of implies that the only way to treat blood pressure is with medication and so there’s nothing about weight, there’s nothing about, there’s no others … especially for our learners, [annotations] would sort of imply “Hey, these are other things that you address besides just prescribing medications.”

— Physician, Design Round 4

“So, I have in the annotation that I stopped the over-the-counter NSAIDs … and then we’re seeing the drop in the blood pressure from that intervention that wouldn’t show up in your med timeline. It pointed out that they were off their meds because they lost their job that year and couldn’t afford their meds.”

— Physician, Design Round 2

#### Data visualization features

Goal ranges indicated by colored bands were well received, rarely questioned for meaning, and physicians appreciated them being set by clinical guidelines. Both groups expressed that clear targets would help them frame their discussion about blood pressure control. Physician participants did indicate the desire to customize the default goal range for appropriate patients.

“I like those little bands [goal ranges] because you can very quickly determine whether your patient is within the designated range … and in addition what proportion of blood pressure measurements are really within that range compared with just looking at a bunch of numbers.”

— Physician, Design Round 2

“For the frail older adult there’s discrepancy about whether 150 is really the right goal, and some would say 160 if you’re not trying to prevent adverse effects in the long term is fine … for me to wiggle that person up to 180 and I’d be happy with that.”

— Physician, Confirmatory Round

“You know, we could then talk about the ranges, which I think makes sense. You know, you’re still at the higher end of this range. We may want to work at getting you into the middle portions.”

— Physician, Confirmatory Round

The design team presented visualizations of annotations that were either physician entered, patient entered, or automatically generated (Fig. 5). Both physicians and patients indicated that the inclusion of informative annotations such as “Lisinopril stopped due to cough” would eliminate repeated searching through the chart and dredging memory for information. Both groups also wanted to be able to annotate the visualization with information that might influence blood pressure control such as “started exercise program” or “increased stress due to father’s illness and daughter’s wedding”; patients and physicians saw great promise in associating this information with blood pressure data to learn the effects of life and lifestyle, similar to the advantage of having the medication timeline as part of the visualization.

However, physicians indicated a concern that their personal effort expenditure for physician-entered annotations might outweigh the benefits of entering annotations manually. They preferred drop-down list options within their existing workflow or automated population of relevant data. They were also concerned about the potential for voluminous patient-entered annotations.

“I like the home entering idea very much, but then like for the few patients that check in like three times a day and put comments on all of them, what do you do about that, I guess? … I get all this information overload and it’s hard to interpret what to do in terms of next steps.”

— Physician, Design Round 4

#### Sensemaking and understanding the blood pressure story

Taken together, the data visualization with goal ranges, medication timeline, and data table allowed physicians and patients to see a snapshot of the patient’s history of hypertension, and promoted a fuller shared understanding of the story of the patient’s medications and blood pressure control.

“Neato … It’s really just creating a story where you see what the blood pressure was, when the medicine was started, where it changed.”

— Physician, Design Round 4

The medication timeline allowed both groups of participants to derive meaning about how changes in medications correlate with changes in blood pressure in a way that might reduce cognitive load. Physicians found the medication timeline to be helpful and mostly user-friendly and they appreciated its flexibility and intuitive nature; both groups believed the tool would alleviate the need to rely on memory or dig through the EHR for historical/contextual notes.

“It shows clearly that when you add, the Hydrochlorothiazide … and then when you added the additional Lisinopril, that looks like the combination of those … made the blood pressure come down.”

— Patient, Design Round 1

“I’ve been through that. My doctor’s like ‘Well, let’s try this one. No, we took you off of it. Do you remember why we took you off of it?’”

— Patient, Design Round 5

**Fig. 6:**
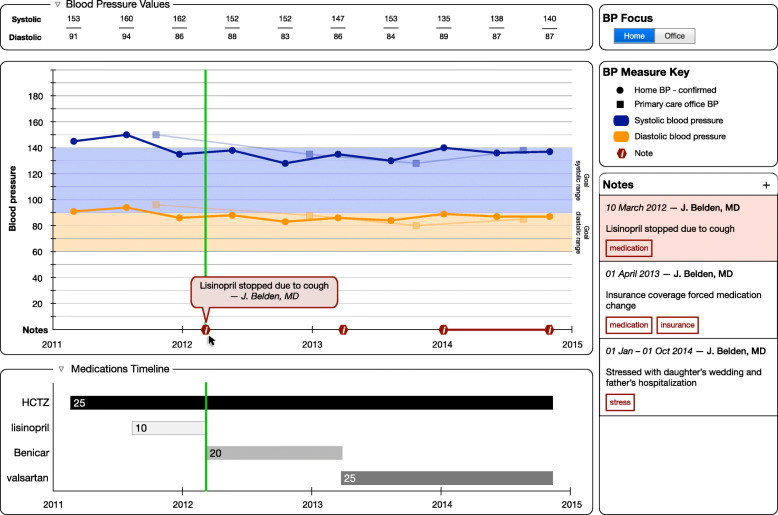
Scenario presented to participants: “You have been working with your doctor to control your blood pressure for a number of years. At one time you stopped taking medication. Discuss”

In particular, both patient and physician participants were able to construct a story of a gap in adherence based on the data visualization shown in Fig. 6, making the connection between the gap in adherence and a rise in blood pressure.

“I mean, it’s doing great … [and then he] quits taking the medication, it’s just out of control.”

— Patient, Design Round 5

Physicians commented that complex medication stories involving other prescribers and transitions of care would be simplified by the medication timeline.

“Some of my patients who are coming in and out of the hospital who are perhaps following with a cardiologist or the nephrologist, sometimes we don’t understand why was this person’s regimen changed and, you know, what led up to that … for people who are, who are, you know, elderly who are having problems or multiple physicians involved with their care it’ll be much more straightforward to see why were these changes made.”

— Physician, Design Round 4

#### Shared understanding of control and goals

Patients and physicians felt this visualization would help them come to consensus on the status of blood pressure control and would better enable them to make treatment decisions. Physicians began to envision how they would use the data visualization in their discussions with patients.

“It’s really not me setting the goal. This is enhancing shared decision making so it’s the patient and I sit down, we define what our goals are, we set those goals, and it’s evidence-based and, and then I define it, and then I continue to say ‘Have we met that goal?’ Just empowering shared decision making rather than me saying I’m setting it.”

— Physician, Confirmatory Round

“I think it’s important for me as a patient to be more intentional and more in, participating and not be so passive with my healthcare.”

— Patient, Confirmatory Round

#### Workflow

Patients and physicians were concerned about workflow, especially collecting and transmitting home blood pressure data from the patient to physician. Use of patient-generated home blood pressure data in the visualization increased the patients’ sense of importance for providing home blood pressures to portray an accurate picture of hypertension control. Physicians anticipated using this data in decision-making at appointments. Both groups suggested that Bluetooth data uploaded from home monitors would ease the patient’s reporting burden.

“What’s really handy for a month or so before you come see me if that’s when you can start tracking your blood pressures again and bring that data because that’s what you’ve done most recently is probably more important.”

— Physician, Confirmatory Round

“The home numbers, are they put in by the physician or is it something that we will put in track as we go?...I take so many blood pressures and my machine only holds so many and then they get wiped out … It would be so convenient just to log into that [patient portal] and type my numbers in once a week or every few days, you know, whenever I do it while it’s fresh on my mind so it’s on there.”

— Patient, Confirmatory Round

Both groups wanted to know more about the flow of entered home blood pressure data and what members of the care team would manage patient home blood pressure data. Both patient and physician participants expressed concerns with home blood pressure data quality and proper measurement technique. Patients and physicians recognized integration of home blood pressure data into a treatment plan would require a new approach in patient care, one that included activated patients and workflow adaptations to accommodate remote monitoring.

“There should be a trigger or something that sends a message to the doctor and it’s just not on the graph and the doctor never sees it until the patient finally comes in again … It’s just sitting there on a graph somewhere in a database and the patient’s getting ready to die (laughs), they’re getting ready to have a serious episode and then when they’re in the ER in the crisis they’ll see all of this and it’s like why didn’t we see a doctor, well, nobody told me.”

— Patient, Confirmatory Round

“I kind of like the idea of having the flag in terms of the [in-person] discussion … but from a population perspective there needs to be something that triggers somebody looking at the data if it has gotten significantly out of line.”

— Physician, Confirmatory Round

## Discussion

We have gained an understanding of primary care physician and patient information needs about blood pressure data for use in their decision-making work for the management of hypertension. We have paid special attention to their needs for home blood pressure information, how they would like to send and receive this information, and their aspirations for using this data in their shared work. Employing a user-centered design process, we synthesized our multidisciplinary expertise with evidence-based knowledge, including cognitive perceptual experiments, and these discovered information needs to create an intuitive and well-received visualization that combines clinical blood pressure data, home blood pressure data, goal ranges, a smoothing algorithm, and a medication timeline [10, 42]. This integration of information would likely increase situational awareness for patients and physicians and has the potential to improve the quality of hypertension care [55]. Eliciting these information needs is a significant contribution to the hypertension, decision support, and EHR design literature, as is our creation and implementation of a user-informed data visualization that includes home blood pressure data in the clinical EHR.

While there is growing evidence for use of home blood pressure data for hypertension decision making [12–18], it is also evident that there are questions about how best to incorporate these data into clinical work, and the physician and care team electronic workflow [19]. While we have created a data visualization as a partial solution to the home blood pressure data problem, work remains to define the flow of home blood pressure data to the care team, including possible alerts for high and low values, but without creating alert fatigue for the clinical team [56, 57]. Defining this data flow and notifications will require significant work and further understanding of user information needs.

While we were inclusive of both patients and physicians, the primary users of these blood pressure data, we did not include another significant stakeholder group, nurses and other members of the patient’s health care team. In addition, our information needs and design work takes a primary care perspective, and we have not considered the needs of specialty physicians. Our rationale for this sampling framework was that the majority of hypertension management occurs in the primary care setting [58]. We have focused on adults with hypertension; pediatric blood pressure data visualization is a more complex design problem as younger children have norms based on both age and height [59]. These current limitations all represent fertile areas for future work. Additionally, patients and physicians attended separate focus groups. We considered the alternative of combining patients and physicians in confirmatory focus groups to create interaction around the designs, however, in addition to being logistically prohibitive among these returning participants, we were also concerned that the presence of physicians in mixed focus groups might effectively diminish the voice of the patient participants, especially if logistics did not allow matching of patients with their own physician.

As a result of this work, we collaborated with our EHR vendor to implement this visualization into the clinical EHR, including patient portal functions that improve patient upload of home blood pressure data and allow it to be represented in the clinical EHR. Thus far, we have not been able to incorporate annotations to the visualization. Work remains for us to identify ways to add annotations that fit into physician workflow, creating opportunities for physicians to add meaningful information easily in a way that will add value to their work. Additionally, we need to understand the best way to add patient annotations, whether to create standardized categories of annotations, and how to deal with a high density of patient annotations. We also need to add Bluetooth capability for home blood pressure devices to the patient portal. Our algorithms are equipped to handle large amounts of blood pressure data, but it is inevitable that Bluetooth upload from devices will introduce new challenges.

A significant next step is to evaluate the ongoing patient and physician use of these tools, not only how much they use these tools, but also how the tools affect the quality and process of care. We will also evaluate how the visualization that includes home blood pressure affects how patients and physicians work together during the outpatient clinic visit to manage hypertension, examining differences between care with and without the visualization with comparison to paper lists of home blood pressures.

Use of these home blood pressure EHR tools will be augmented by two new environmental factors. First, the January 2020 introduction of new Medicare-reimbursed current procedural terminology (CPT) codes for home blood pressure management activities could drive adoption [60]. Second, the significantly increased use of telehealth due to the COVID-19 pandemic can leverage the home blood pressure data visualization into an opportunity to maintain quality of care for patients with hypertension during a time of social distancing and increased reliance on home blood pressure data [61].

## Conclusions

Our blood pressure data visualization that includes home blood pressure data is a significant innovation that has potential to improve the quality of care for patients with hypertension, both through a better patient-physician understanding of blood pressure data, and in creating new ways for patients and physicians to interact around these data in their decision making process during outpatient visits. The patient’s active participation in generating these data might better engage them in the management process and be more convincing for those who resist blood pressure medication intensification by facilitating the “prepared patient and proactive health care team” that are at the heart of the productive interactions that lead to improved outcomes in the Chronic Care Model [38, 39].

## Data Availability

The data that support the findings of this study are available from the corresponding author, RJK, upon reasonable request.

## Abbreviation

EHR: Electronic Health Record

## References

[1] Agency for Healthcare Research and Quality (2019). Adults with hypertension with blood pressure less than 140/90 mm/Hg, United States, 1999-2014.

[2] Go AS, Mozaffarian D, Roger VL, Benjamin EJ, Berry JD, Borden WB (2013). Heart disease and stroke statistics--2013 update: a report from the American Heart Association. Circulation..

[3] Benjamin Emelia J, Virani Salim S, Callaway Clifton W, Chamberlain Alanna M, Chang Alexander R, Cheng S (2018). Heart disease and stroke statistics—2018 update: a report from the American Heart Association. Circulation..

[4] Phillips LS, Branch WT, Cook CB, Doyle JP, El-Kebbi IM, Gallina DL (2001). Clinical inertia. Ann Intern Med.

[5] O'Connor PJ (2003). Overcome clinical inertia to control systolic blood pressure. Arch Intern Med.

[6] Okonofua EC, Simpson KN, Jesri A, Rehman SU, Durkalski VL, Egan BM (2006). Therapeutic inertia is an impediment to achieving the healthy people 2010 blood pressure control goals. Hypertension..

[7] Kerr EA, Zikmund-Fisher BJ, Klamerus ML, Subramanian U, Hogan MM, Hofer TP (2008). The role of clinical uncertainty in treatment decisions for diabetic patients with uncontrolled blood pressure. Ann Intern Med.

[8] Parati G, Stergiou GS, Asmar R, Bilo G, De LP, Imai Y (2008). European Society of Hypertension guidelines for blood pressure monitoring at home: a summary report of the second international consensus conference on home blood pressure monitoring. J Hypertens.

[9] Parati G, Tortorici E, Glavina F, Zaniboni D, Gritti S, Groppelli A (2001). Blood pressure variability. Ital Heart J Suppl.

[10] Shaffer VA, Wegier P, Valentine K, Belden JL, Canfield SM, Patil SJ (2019). Patient judgments about hypertension control: the role of variability, trends, and outliers in visualized blood pressure data. J Med Internet Res.

[11] Kolata GL (2015). Blood pressure, a Reading with a habit of straying.

[12] Uhlig K, Patel K, Ip S, Kitsios GD, Balk EM (2013). Self-measured blood pressure monitoring in the management of hypertension: a systematic review and meta-analysis. Ann Intern Med.

[13] Ohkubo T, Imai Y, Tsuji I, Nagai K, Kato J, Kikuchi N (1998). Home blood pressure measurement has a stronger predictive power for mortality than does screening blood pressure measurement: a population-based observation in Ohasama. Japan J Hypertens.

[14] Bobrie G, Chatellier G, Genes N, Clerson P, Vaur L, Vaisse B (2004). Cardiovascular prognosis of "masked hypertension" detected by blood pressure self-measurement in elderly treated hypertensive patients. JAMA..

[15] Sega R, Facchetti R, Bombelli M, Cesana G, Corrao G, Grassi G (2005). Prognostic value of ambulatory and home blood pressures compared with office blood pressure in the general population: follow-up results from the Pressioni Arteriose Monitorate e Loro Associazioni (PAMELA) study. Circulation..

[16] Fagard RH, Van Den Broeke C, De CP (2005). Prognostic significance of blood pressure measured in the office, at home and during ambulatory monitoring in older patients in general practice. J Hum Hypertens.

[17] Oikawa T, Obara T, Ohkubo T, Kikuya M, Asayama K, Metoki H (2006). Characteristics of resistant hypertension determined by self-measured blood pressure at home and office blood pressure measurements: the J-HOME study. J Hypertens.

[18] Pickering TG, Miller NH, Ogedegbe G, Krakoff LR, Artinian NT, Goff D (2008). Call to action on use and reimbursement for home blood pressure monitoring: a joint scientific statement from the American Heart Association, American society of hypertension, and preventive cardiovascular nurses association. Hypertension..

[19] Koopman RJ, Wakefield BJ, Johanning JL, Keplinger LE, Kruse RL, Bomar M, et al. Implementing home blood glucose and blood pressure telemonitoring in primary care practices for patients with diabetes: lessons learned. Telemed J E Health. 2014;20(3):253–60. 10.1089/tmj.2013.0188. 10.1089/tmj.2013.0188 PMC3934548 24350806

[20] Barr VJ, Robinson S, Marin-Link B, Underhill L, Dotts A, Ravensdale D (2003). The expanded chronic care model: an integration of concepts and strategies from population health promotion and the chronic care model. Hosp Q.

[21] Kadu MK, Stolee P (2015). Facilitators and barriers of implementing the chronic care model in primary care: a systematic review. BMC Fam Pract.

[22] Wagner EH, Austin BT, Davis C, Hindmarsh M, Schaefer J, Bonomi A (2001). Improving chronic illness care: translating evidence into action. Health Aff (Millwood ).

[23] Holmes G (1984). How to present your message graphically. Accountancy..

[24] Kosslyn SM (2006). Graph design for the eye and mind.

[25] Bauer DT, Guerlain S, Brown PJ (2010). The design and evaluation of a graphical display for laboratory data. J Am Med Inform Assoc.

[26] Brewer NT, Gilkey MB, Lillie SE, Hesse BW, Sheridan SL (2012). Tables or bar graphs? Presenting test results in electronic medical records. Med Decis Mak.

[27] Koopman RJ, Kochendorfer KM, Moore JL, Mehr DR, Wakefield DS, Yadamsuren B (2011). A diabetes dashboard and physician efficiency and accuracy in accessing data needed for high-quality diabetes care. Ann Fam Med.

[28] Tufte ER (2001). The visual display of quantitative information.

[29] Hawley ST, Zikmund-Fisher B, Ubel P, Jancovic A, Lucas T, Fagerlin A (2008). The impact of the format of graphical presentation on health-related knowledge and treatment choices. Patient Educ Couns.

[30] McCaffery KJ, Dixon A, Hayen A, Jansen J, Smith S, Simpson JM (2012). The influence of graphic display format on the interpretations of quantitative risk information among adults with lower education and literacy: a randomized experimental study. Med Decis Mak.

[31] Kabutoya T, Ishikawa J, Hoshide S, Eguchi K, Shimada K, Kario K (2009). A home blood pressure monitor equipped with a graphic function facilitates faster blood pressure control than the conventional home blood pressure monitor. J Clin Hypertens.

[32] Cleveland WS, McGill R (1984). Graphical perception: theory, experimentation, and application to the development of graphical methods. J Am Stat Assoc.

[33] Johnson JR, Rice RR, Roemmich RA (1980). Pictures that lie: the abuse of graphs in annual reports. Manag Account.

[34] Belden J, Patel J, Lowrance N, Plaisant C, Koopman R, Moore J, et al. Inspired EHRs: designing for clinicians: Curators of the University of Missouri; 2014. Available at https://inspiredehrs.org/.

[35] Abras C, Maloney-Krichmar D, Preece J (2004). User-centered design. Bainbridge, W Encyclopedia of Human-Computer Interaction.

[36] Nielsen J. Usability engineering. Cambridge: Academic Press; 1993.

[37] Ratwani RM, Fairbanks RJ, Hettinger AZ, Benda NC (2015). Electronic health record usability: analysis of the user-centered design processes of eleven electronic health record vendors. J Am Med Inform Assoc.

[38] Barr V, Robinson S, Marin-Link B, Underhill L, Dotts A, Ravensdale D (2003). The expanded chronic care model. Hosp Q..

[39] Wagner EH, Davis C, Schaefer J, Von Korff M (1999). A survey of leading chronic disease management programs: are they. Managed Care Quart.

[40] Witteman HO, Stahl JE (2013). Facilitating interdisciplinary collaboration to tackle complex problems in health care: report from an exploratory workshop. Health Syst.

[41] Makoul G, Clayman ML (2006). An integrative model of shared decision making in medical encounters. Patient Educ Couns.

[42] Belden JL, Wegier P, Patel J, Hutson A, Plaisant C, Moore JL (2018). Designing a medication timeline for patients and physicians. J Am Med Inform Assoc.

[43] Gill P, Stewart K, Treasure E, Chadwick B (2008). Methods of data collection in qualitative research: interviews and focus groups. Br Dent J.

[44] Beebe J. Rapid assessment process: an introduction. Walnut Creek: Rowman Altamira Press; 2001.

[45] Palinkas LA, Zatzick D (2019). Rapid assessment procedure informed clinical ethnography (RAPICE) in pragmatic clinical trials of mental health services implementation: methods and applied case study. Adm Policy Ment Health Ment Health Serv Res.

[46] Taylor B, Henshall C, Kenyon S, Litchfield I, Greenfield S (2018). Can rapid approaches to qualitative analysis deliver timely, valid findings to clinical leaders? A mixed methods study comparing rapid and thematic analysis. BMJ Open.

[47] Braun V, Clarke V (2006). Using thematic analysis in psychology. Qualitative research in psychology. Qual Res Psychol.

[48] Wakefield DS, Mehr D, Keplinger L, Canfield S, Gopidi R, Wakefield BJ (2010). Issues and questions to consider in implementing secure electronic patient–provider web portal communications systems. Int J Med Inform.

[49] Clarke MA, Moore JL, Steege LM, Koopman RJ, Belden JL, Canfield SM (2018). Toward a patient-centered ambulatory after-visit summary: identifying primary care patients’ information needs. Inf Health Social Care.

[50] Koopman RJ, Steege LMB, Moore JL, Clarke MA, Canfield SM, Kim MS (2015). Physician information needs and electronic health records (EHRs): time to reengineer the clinic note. J Am Board Fam Med.

[51] Clarke MA, Belden JL, Koopman RJ, Steege L, Moore J, Canfield S, et al., editors. Creating a more readable electronic health record (EHR) model: analysis of primary care physicians' information needs. Chicago. American Medical Informatics Association; 2012.

[52] Shigaki CL, Koopman RJ, Kabel AM, Canfield SM. Successful weight loss: how information technology is used to lose. Telemed J E Health.2014;20(2):144–51. 10.1089/tmj.2013.0163. 10.1089/tmj.2013.0163 24303931

[53] Clarke MA, Belden JL, Koopman RJ, Steege LM, Moore JL, Canfield SM, et al. Information needs and information-seeking behaviour analysis of primary care physicians and nurses: a literature review. Health Info Libr J. 2013;30(3):178–90. 10.1111/hir.12036. 10.1111/hir.12036 23981019

[54] Dedoose (2018). Version 8.0. Web application for managing, analyzing, and presenting qualitative and mixed method research data.

[55] Endsley MR. Designing for situation awareness: an approach to user-centered design: CRC press; 2016.

[56] Gregory ME, Russo E, Singh H (2017). Electronic health record alert-related workload as a predictor of burnout in primary care providers. Appl Clin Inform.

[57] Ancker JS, Edwards A, Nosal S, Hauser D, Mauer E, Kaushal R (2017). Effects of workload, work complexity, and repeated alerts on alert fatigue in a clinical decision support system. BMC Med Inf Dec Making.

[58] Fang J, Alderman MH, Keenan NL, Ayala C, Croft JB (2008). Hypertension control at Physicians' offices in the United States. Am J Hypertens.

[59] Flynn JT, Kaelber DC, Baker-Smith CM, Blowey D, Carroll AE, Daniels SR (2017). Clinical practice guideline for screening and management of high blood pressure in children and adolescents. Pediatrics..

[60] Association AH (2020). New CPT Codes to Cover Self-Measures Blood Pressure (SMBP).

[61] Wosik J, Fudim M, Cameron B, Gellad ZF, Cho A, Phinney D, et al. Telehealth transformation: COVID-19 and the rise of virtual care. J Am Med Inform Assoc. 2020;27(6):957–962. 10.1093/jamia/ocaa067. 10.1093/jamia/ocaa067 PMC7188147 32311034

